# An Illustrated System of Human Anatomy, Special, General, and Microscopic

**Published:** 1849

**Authors:** 


### ARTICLE V.

*An Illustrated System of Human Anatomy, Special, General, and Microscopic.* By SAMUEL GEORGE MORTON, M. D., Penn. and Edinb., Member of the Medical Societies of Philadelphia, New York, Boston, Edinburgh, and Stockholm, author of "Crania Americana," "Crania Ægyptiaca," etc. With three hundred and ninety-one engravings on wood. "Oculis subjecta fidelibus." Philadelphia: Grigg, Elliott, & Co., No. 14 North Fourth Street. 1849. Royal 8vo, pp. 642. (From the publishers.)

The prominent and most active medical men and writers of the present time may very properly be classed as follows: into hard working, sensible truth seekers; credulous, imaginative theorisers; and experimentalists; each furnishing, from time to time, in books, pamphlets, and medical periodicals, matter adapted to the tastes and talents of every class of minds.

The work before us, by the justly celebrated author of the "Crania Americana," "Crania Ægyptiaca," etc., Dr. Morton, is a treatise upon one of the most important branches of the science of medicine, written in a chaste and concise style, superior to any work of the kind ever published in this country in its typographical execution and in the correctness of its illustrations, and is, in every respect, adapted to please and interest that best class of medical men who endeavor to base their knowledge and rules of practice upon well-known anatomical, pathological, and physiologial facts, rather than upon the visionary speculations of those who, under the garb of science, promulgate opinions and doctrines no less absurd in some instances, than those of the infinitesimal and eclectic schools.

The work adds additional lustre to the well earned reputation of the author, and is got up in a style which shows most conclusively that our American pablishers, when sufficient encouragement shall be offered them, can furnish us books in every respect equal, if not superior, in quality of paper, style of plates, and typography, to the illustrated works, now, for the most part, obtained from abroad.

The nature of the work is such, that it would be difficult, if not impossible, to give an abstract of its contents in the small space in our journal allotted to bibliographical notices; we must, therefore, deny ourselves the pleasure we always take in noticing at large medical productions of this class, and say, in conclusion, that Dr. Morton's Illustrated Anatomy deserves a place in the medical library of every American physician and student, both on account of the intrinsic value of the work, and for the no less important consideration that it is the duty of the profession to encourage the talents and skill of American authors and publishers.

For the reasons given above, it will be recommended to students and adopted as a text book on anatomy in Rush Medical College. H.

